# Anterior mediastinal metastasis of primary fallopian tube adenocarcinoma: a case report

**DOI:** 10.1186/s13019-020-01111-4

**Published:** 2020-05-11

**Authors:** Bo Zhang, Renwang Liu, Tong Li, Feng Chen, Huandong Huo, Dian Ren, Fan Ren, Song Xu, Xiaohong Xu, Zuoqing Song

**Affiliations:** 1 Department of Lung Cancer Surgery, Tianjin Medical University General Hospital, Anshan Road No 154, Heping District, Tianjin, 300052 China; 2 Department of Lung Cancer Surgery; Tianjin key laboratory of lung cancer metastasis and tumor microenvironment, Tianjin Lung Cancer Institute, Tianjin Medical University General Hospital, Anshan Road No.154, Heping District, Tianjin, 300052 China; 3 Colleges of Nursing, Tianjin Medical University, Qixiangtai Road No.22, Tianjin, 300070 China

**Keywords:** Anterior mediastinal metastasis, Primary fallopian tube carcinoma (PFTC), Resection

## Abstract

**Background:**

Primary fallopian tube carcinoma (PFTC) is a malignant tumor of the female genital tract that mostly presents intraperitoneal dissemination in clinical practice. The incidence of upper anterior mediastinal metastasis in PFTCs is extremely rare. We herein report a rare case of PFTC mediastinal metastasis after radical resection. When anterior mediastinal metastasis of an unknown origin is encountered, the possibility of PFTC should be considered.

**Case presentation:**

A 68-year-old female who was previously diagnosed with PFTC after radical resection of a primary tumor in the fallopian tube was admitted to our department with a right anterior mediastinum mass. Radical resection of the mediastinal mass was performed, and poorly differentiated metastatic adenocarcinoma of the fallopian tube was confirmed. There was no recurrence in the 24 months after the curative operation.

**Conclusion:**

To our knowledge, no mediastinal metastasis of PFTC has been reported yet. Thus, we presented this rare case indicating the heterogeneity of this malignant disease and to draw attention to the occasional distant metastasis of PFTC in clinical practice.

## Introduction

Primary fallopian tube carcinoma (PFTC) is an aggressive, malignant tumor of the female genital tract with an unfavorable prognosis. It is often classified with primary ovarian and peritoneal carcinomas within the epithelial ovarian cancer umbrella. There are currently at least five major histologic types of epithelial ovarian cancer: high- and low-grade serous, endometrioid, mucinous, and clear cell carcinoma. High-grade serous carcinomas are the most common type, comprising 70–80% of cases. More recent evidence has suggested that epithelial ovarian cancers originate from a fallopian tube precursor. When grouped together, ovarian, fallopian tube and primary peritoneal carcinomas account for 2.5% of all new female cancer cases in the United States (an incidence rate of 11.6 per 100,000 women per year, amounting to more than 22,000 diagnoses each year) [1, 2], which may account for the high incidence of PFTC. There are also many factors that have led to the incidence of PFTC increasing by 4.19-fold from 2001 to 2014, including changes in diagnostic practices, increased early detection, and improved pathology processing [3, 4]. Usach et al. showed that PFTC had better overall survival than ovarian and primary peritoneal carcinomas (50% versus 37 and 40%, respectively) [5]. The pathways of metastasis of PFTC include hematogenous and lymphatic metastasis, peritoneal transcoelomic spread, direct invasion, etc. Intraperitoneal metastasis, including lymph node metastasis, peritoneal transcoelomic spread and regional invasion, is the major metastasis pathways in this disease. However, extra-abdominal metastasis in PFTC is extremely rare, as is mediastinal metastasis. Here, to the best of our knowledge, we report a rare case of PFTC mediastinal metastasis for the first time.

## Case presentation

**Fig. 1:**
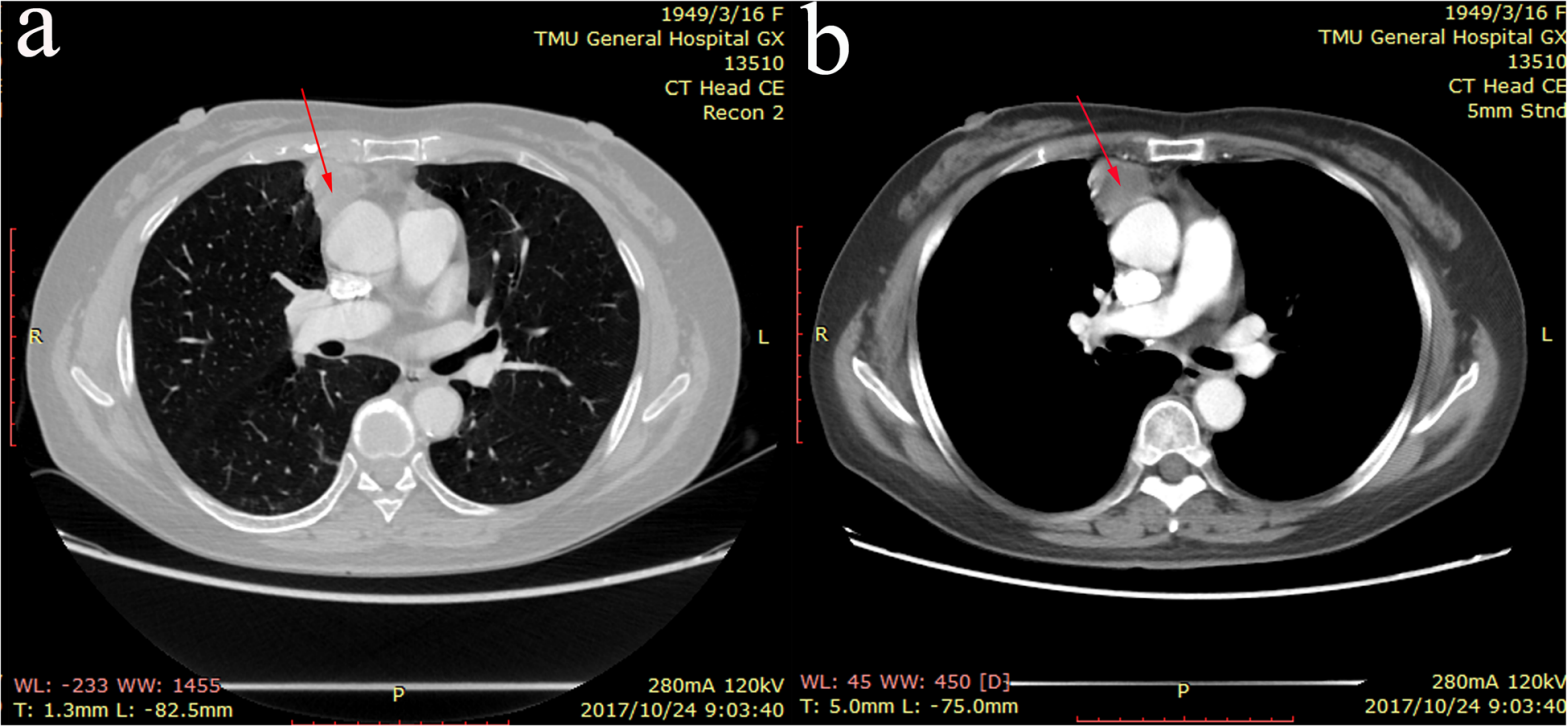
Chest CT scans. Chest CT scans of this patient showed a 27 mm × 28 mm (arrows) mass with mixed cystic solid lump, irregular lobes and fat density features in right upper mediastinum

**Fig. 2:**
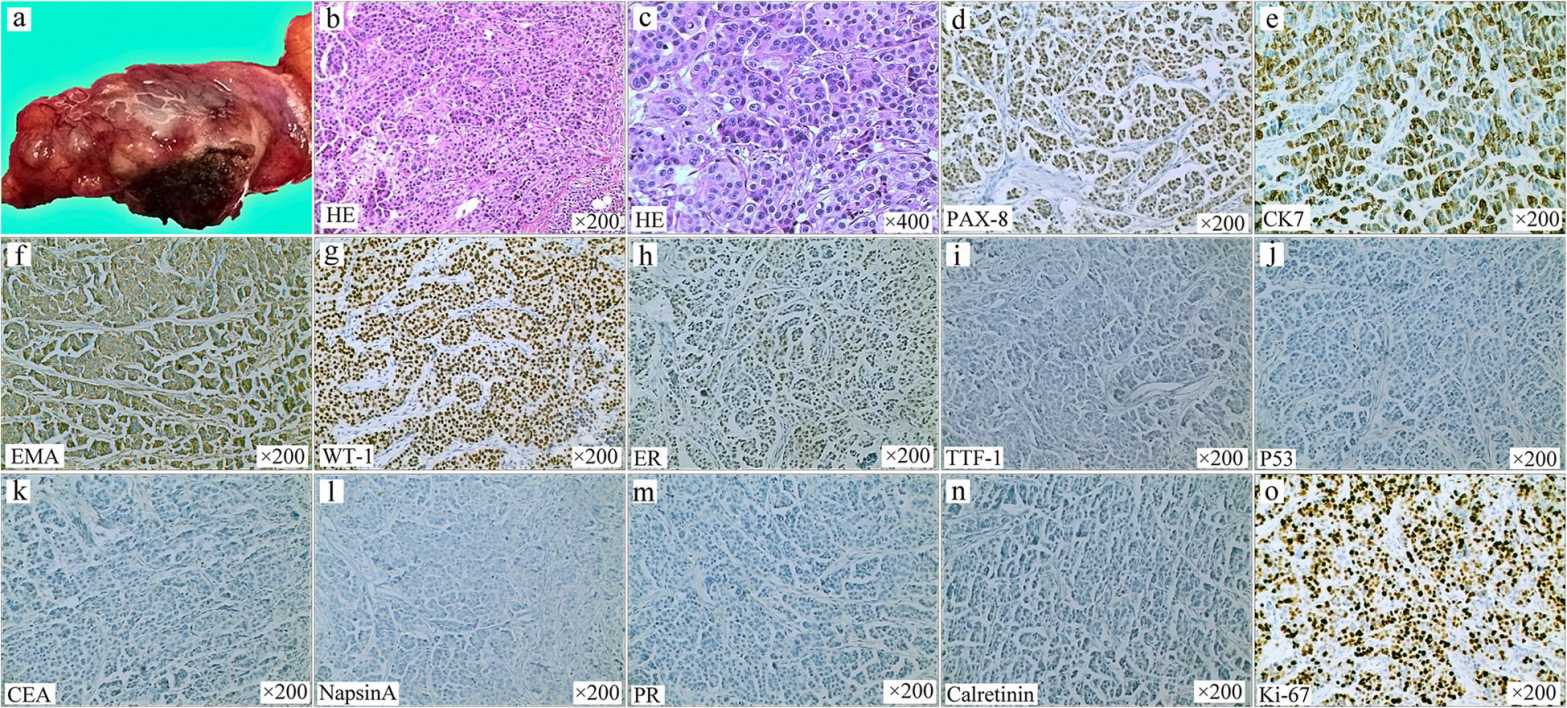
The pathological characters of the tumor. **a** Pathological finding of the mediastinal irregular mass. **b** - **c** microscopically, hematoxylin-eosin (HE) staining showed serous carcinoma with the cells had large, oval-shaped and deeply stained nuclei. **d** - **h** Immunohistochemical (IHC) staining of the tumor with antibodies to PAX-8, CK7, EMA and WT-I were strong positive expression, and ER partly positive expression, and **i** – **n** negative expression of P53, CEA, NapsinA, PR, Calretinin and TTF-1, respectively. **o** the Ki-67 labeling index was 70%

A 68-year-old postmenopausal female was admitted to our department for a mediastinal mass that was found via chest computed tomography (CT). She was diagnosed with low differentiated adenocarcinoma of the fallopian tube without regional lymphatic metastasis, including the retroperitoneal perivascular and pelvic mesenteric areas and the right iliac blood vessel, after radical resection, including uterine, bilateral attachment and pelvic lymph node dissection, was performed in another hospital 5 years before. Five cycles of chemotherapy with a paclitaxel and cisplatin (TP) regimen were subsequently administered to this patient. None of the significant symptoms presented in the previous 5 years of routine follow-up until a mediastinal mass was found on CT scan, which was performed because of a cough with expectoration that presented in the patient 10 days earlier. There was no pertinent family history of cancer. Her physical examination had no obvious abnormalities. The enhanced chest CT showed a mixed cystic-solid mass with irregular lobes and fat density in the right upper mediastinum, approximately 27 mm × 28 mm in size (Fig. 1). No evidence of metastasis was found in the upper abdomen, brain or bone via enhanced CT, magnetic resonance imaging (MRI) or single-photon emission computed tomography (SPECT). Serum testing of tumor markers showed that the cancer antigen 125 (CA-125) level was 99.00 U/ml (normal, < 35 U/ml), and the cytokeratin-19 fragment (CYFRA21-1) level was 4.09 ng/ml (normal, < 3.3 ng/ml). Carcinoembryonic antigen (CEA), CA153, neuron-specific enolase (NSE) and squamous cell carcinoma antigen (SCC) levels were normal in this patient. Resection of the right upper mediastinal mass, mediastinal lymph node dissection and anterior mediastinal fat dissection were performed via video-assisted thoracoscopic surgery (VATS), as the diagnosis of primary malignant mediastinal tumor was considered priority. Because of the tumor in the right upper mediastinum, we chose the lateral intercostal approach. The patient was placed in the left lateral decubitus position. The incision was made in the fourth right intercostal space at the anterior axillary line. The tumor was to the front of the ascending aorta. To ensure en bloc dissection of the tumor, we excised the tumor with an ultrasonic scalpel, not with an electric hook. The dissection started at the anterior border of the phrenic nerve. The tumor was then mobilized to expose the ascending aorta and innominate vein. Then, the mediastinal fat was fully dissected from the phrenic nerve, the aortopulmonary window, and the aorta. The mass was radically resected (Fig. 2a). microscopically, hematoxylin-eosin (HE) staining showed that the mediastinal tumor was composed of serous adenocarcinoma cells (Fig. 2b - c), and mediastinal metastasis of the PFTC was confirmed by strong positive expression of PAX-8, CK7, EMA and WT-1, partial positive expression of estrogen receptor and negative expression of P53, CEA, Napsin A, PR, calretinin and TTF-1, as verified by immunohistochemical (IHC) staining (Fig. 2d - n). The Ki-67 labeling index was 70% (Fig. 2o), and one of four lymph nodes in the adipose tissue surrounding the tumor was also demonstrated to contain metastasis in this patient. Six cycles of the TP regimen were performed after resection. The patient was followed up regularly by head MRI and enhanced chest and whole abdominal CT scans. There are no signs of recurrence or metastasis to date.

## Discussion

Mediastinal tumors, including primary tumors and secondary tumors, are common in the chest and are most commonly thymomas and retrosternal thyroid tumors. CT can not only show the size, density and edge of the tumor but also indicate the relationship between the tumor and the surrounding organs in the chest, including large vessels, lungs, pericardium, heart, pleura, etc. However, it is very difficult to distinguish primary and second tumors by chest CT. Metastatic mediastinal tumors occur as a result of tumor cell invasion originating from an adjacent organ and pleural and distant metastasis. Nevertheless, mediastinal metastasis from fallopian tube carcinoma is extremely uncommon. Metastasis of this disease has been reported in recent years. The rare and distant metastasis sites of PFTC include supradiaphragmatic lymph nodes, axillary lymph nodes, brain, vagina, femur and rib, ovary, cervix, lung, spine, colon, skin and diaphragm [6–21]. However, to our knowledge, no mediastinal metastasis of this tumor has been reported yet. Hence, we report for the first time a rare case of PFTC mediastinal metastasis.

PFTCs occur most frequently in patients of 55–60 years [22]. The etiology of this tumor is still unknown, and because such cancer types tend to be diagnosed at advanced stages, the early molecular events underlying development are not fully clarified. A series of studies may attribute such cases to chronic tubal inflammation, infertility, tuberculosis salpingitis or tubal endometriosis [23]. Meanwhile, germline BRCA1 and BRCA2 mutations, as in ovarian and breast cancer, showed an increased tendency to lead to PFTC development [24–27]. PFTCs usually originate in the ampulla, and the progression pattern can be nodular, papillary, and infiltrative [10]. The majority of symptoms of PFTCs fall within a set of symptoms called the Latzko triad, including pelvic pain, pelvic mass, and serosanguinous vaginal discharge.

The most common pathway of PFTC metastasis, occurring in 80% of cases (similar to the occurrence in ovarian cancer), is intraperitoneal dissemination, including lymph node metastasis, uterus invasion and ovarian invasion [28]. M.D. et al. demonstrated that intraperitoneal lymph node metastasis of PFTC accounted for 59% of para-aortic lymph node lesions, accounting for 33% of cases [29]. Fully understanding the lymphatic drainage from the fallopian tube may shed light on the metastatic pattern. The proximal portion of the tube closest to the uterus drains towards the para-aortic nodes, and the distal portion including the fimbriae drains towards the pelvic nodes, leading to a favorable metastatic site for primary tumors in each region [29–31]. Other rare metastatic sites have also been reported. Skin metastases are rare and are classified as umbilical metastasis (Sister Mary Joseph’s nodule [SJN]) and nonumbilical metastasis. Fifty-three percent of Sister Mary Joseph’s nodules originated from malignancies of the female genital tract, particularly ovarian carcinoma [20]. Jonathan P. Eskander et al. reported a case of a 68-year-old woman with metastasis of a fallopian tube cancer to the L1 vertebral body [17]. A 50-year-old woman with metastasis of a fallopian tube cancer to the ovary has also been reported [15]. Other uncommon metastatic regions, including the central nervous system (CNS), uterus and diaphragm, have also been reported recently [11, 21, 32]. The reported metastatic regions of PFTC are summarized in Table 1. Nevertheless, mediastinal metastasis of PFTC has not yet been reported. This suggests that nonpulmonary metastases alone as the sole form of distant (extra-abdominal) metastatic spread are very unusual. The exact mechanism by which cancer cells metastasize to the upper anterior mediastinum from fallopian tube malignancy is unclear. Even though the main pattern of metastatic spread of PFTC occurs via lymphatic vessels, hematogenous spread to the site cannot be excluded. Hematogenous dissemination plays a critical role in the distant metastasis of epithelial ovarian cancer [33]. Further research may focus on the mechanism underlying the mediastinal metastasis of PFTC. Given that its clinical behavior is similar to that of epithelial ovarian cancer, PFTC is managed in the same way: debulking surgery and platinum-based chemotherapy. Novel targeted therapies may extend the survival of patients with advanced fallopian tube carcinoma, including poly (adenosine diphosphate [ADP]-ribose) polymerase (PARP) inhibitors, anti-vascular endothelial growth factor (VEGF) antibodies and programmed cell death protein [PD]-1 inhibitors. PARP inhibitors are particularly efficacious in patients with BRCA1/2 gene mutations [34] and have been shown to benefit patients. Here, we reported this rare case to alert clinicians to the potential that PFTC may present mediastinal metastasis occasionally in clinical practice. For patients with PFTC at an advanced stage, it is necessary to perform aggressive cytoreductive surgery [35].

**Table 1:** Literature review of metastasis of PFTC

Author	Age	Metastatic site	Sizes of metast-atic site (mm)	Symptoms	Treatment	Metastasis of pelvic, and/or para-aortic lymph node
	(years)					
Harl F et al. [11]	68	CNS	N/A	Mild confusion and anomic aphasia	MTR, HC, BSO, RT, CT	left para-aortic lymph node
Qinhe Zhang et al. [16]	49	cervix	9 × 5	Lower abdominal pain and colporrhagia	HC, BA, PLD, OME, CT	none
Toyoda T et al. [21]	83	diaphragm	30	Physical examination discovery	HC, BSO, LAR-R, IPSSO, CT	N/A
Eken MK et al. [7]	60	Left supra-clavicular lymph node	N/A	Palpable left supraclavicular lymph node	HC, BSO, AE, T-O, PPALE, CT	none
Eskander JP et al. [17]	68	Spinal	N/A	Intractable back pain and lower extremity weakness	L1 corpectomy and reconstruct-ion, CT, RT	N/A
Courville et al. [13]	56	right femur and left eighth anterior rib	N/A	Evaluation of a	CT, RT	N/A
				right proximal femur lesion		
Atallah C et al. [9]	73	right axillary lymph nodes	30	Palpable enlarged right axillary lymph	HC, SO	N/A
				nodes		
Guler I et al. [8]	61	axillary	N/A	Palpable left axillary masspalpable left axillary mass	HC, BSO, PPA-LE, OE, SSCR	four
						metastatic pelvic lymph nodes
Kadour-Peero E et al. [12]	41	vaginal Mass	100	Irregular vaginal bleeding, vaginal mucous discharge and suspected pelvic mass	NCT, HC, BSO, OE, partial vaginectomy, ARR, LND	two metastatic lymph nodes
Wah N et al. [15]	50	ovarian	30 × 20 × 10	The ill-defined tender mass and intermittent bleeding per vaginum	HC, BSO	N/A
Usui G et al. [18]	65	colon	65 × 29	Constipation and diarrhea	NCT, HC, BSO	para-aortic and mesen-
						teric lymph nodes
Kirshtein B et al. [19]	54	umbilical	15 × 10 × 7	Umbilical hernia	HC, OE, CT	N/A
Winter-Roach BA et al. [30]	69	right inguinal lymph node	15	Right-side inguinal swelling	HC, BSO, OE, P-PALE, CT	one right pelvic and one para-aortic lymph node

*CNS* central nervous system, *MTR* microsurgical tumor removal, *HC* hysterectomy, *RT* radiotherapy, *CT* chemotherapy, *BSO* bilateral salpingo-oophorectomy, *BA* bilateral adnexectomy, *PLD* pelvic lymph node dissection, *OME* omentum majus excision, *LARR* low anterior resection of the rectum, *IPSSO* ileocecal peritoneal stripping and subtotal omentectomy, *AE* appendectomy, *TO* total omentectomy, *PPALE* pelvic para-aortic lymphadenectomy, *OE* omentectomy, *SO* salpingo-oophorectomy, *SSCR* egmentary sigmoid colon resection, *NCT* neoadjuvant chemotherapy, *ARR* anterior rectal resection, *LND* lymph node dissection

## Conclusion

PFTC is a rare disease, the most common metastasis region of which is intraperitoneal areas. Here, to the best of our knowledge, we report a case of PFTC with mediastinal metastasis for the first time, which might indicate the heterogeneity of this disease. Despite the extremely rare incidence of mediastinal metastasis of PFTC, the occasional distant metastasis of PFTC may still be seen in clinical practice. Meanwhile, as the mechanism underlying this disease is unclear, further study may focus on it in the future.

## Data Availability

All data generated or analyzed are included in this article.

## Abbreviations

CT: Computed tomography
SPECT: Single-photon emission computed tomography
PFTC: Primary fallopian tube carcinoma
HE: Hematoxylin-eosin
IHC: Immunohistochemically

## References

[1] Siegel RL, Miller KD, Jemal A (2018). Cancer statistics, 2018. CA Cancer J Clin.

[2] Tolia M, Tsoukalas N, Sofoudis C (2016). Primary extramammary invasive Paget’s vulvar disease: what is the standard, what are the challenges and what is the future for radiotherapy. BMC Cancer.

[3] Liao CI, Chow S, Chen LM, Kapp DS, Mann A, Chan JK (2018). Trends in the incidence of serous fallopian tube, ovarian, and peritoneal cancer in the US. Gynecol Oncol.

[4] Trabert B, Coburn SB, Mariani A (2018). Reported incidence and survival of fallopian tube carcinomas: a population-based analysis from the North American Association of Central Cancer Registries. J Natl Cancer Inst.

[5] Usach I, Blansit K, Chen LM (2015). Survival differences in women with serous tubal, ovarian, peritoneal, and uterine carcinomas. Am J Obstet Gynecol.

[6] Klein M, Rosen A, Lahousen M (1994). Lymphogenous metastasis in the primary carcinoma of the fallopian tube. Gynecol Oncol.

[7] Eken MK, Kaygusuz EI, Temizkan O, İlhan G, Çöğendez E, Karateke A (2016). Occult serous carcinoma of fallopian tube presenting as supraclavicular lymphadenopathy. Taiwan J Obstet Gynecol.

[8] Guler I, Onan AM, Hatipoglu O, Taskiran C, Uner A, Guner H (2014). Carcinoma of the fallopian tube presenting as an axillary palpable mass. J Obstet Gynaecol.

[9] Atallah C, Altinel G, Fu L, Arseneau J, Omeroglu A (2014). Axillary metastasis from an occult tubal serous carcinoma in a patient with ipsilateral breast carcinoma: a potential diagnostic pitfall. Case Rep Pathol.

[10] Jayashree K, Anubuti C, Gundappa M (2009). Primary fallopian tube adenocarcinoma with brain and lung metastasis. Indian J Pathol Microbiol.

[11] Harl F, Niemi C, Mankowski GL, Weisman P, Rose S (2018). Solitary CNS metastasis on initial presentation of high grade serous carcinoma of the fallopian tube. Case Rep Obstet Gynecol.

[12] Kadour-Peero E, Sagi-Dain L, Cohen G (2018). Primary papillary serous carcinoma of the fallopian tube presenting as a vaginal mass: a case report and review of the literature. Am J Case Rep.

[13] Courville XF, Cortés Z, Katzman PJ, Rosier RN (2005). Case report: bone metastases from fallopian tube carcinoma. Clin Orthop Relat Res.

[14] Aich RK, Dasgupta S, Chakraborty B, Karim R, Bhattacharya J, Sen P (2012). Primary fallopian tube carcinoma with metastasis in the contralateral ovary. J Indian Med Assoc.

[15] Marwah N, Garg M, Garg S, Sethi D (2012). Primary papillary adenocarcinoma of the fallopian tube with ovarian metastasis. J Lab Physicians.

[16] Zhang Q, Liu A, Wu JJ (2018). Primary malignant mixed Müllerian tumors of the fallopian tube with cervix metastasis: a rare case report and literature review. Medicine (Baltimore).

[17] Eskander JP, Kuris EO, Younghein AJ, Landsman S, Japko L, Eskander MS (2015). Spinal metastases from a primary fallopian tube serous adenocarcinoma: a case report. Global Spine J.

[18] Usui G, Masuda Y, Hashimoto H (2019). Colon metastasis from microscopic serous carcinoma of the fallopian tube fimbria mimicking a primary colon cancer. Int J Surg Pathol.

[19] Kirshtein B, Meirovitz M, Okon E, Piura B (2006). Sister Mary Joseph’s nodule as the first presenting sign of primary fallopian tube adenocarcinoma. J Minim Invasive Gynecol.

[20] Dubreuil A, Dompmartin A, Barjot P, Louvet S, Leroy D (1998). Umbilical metastasis or Sister Mary Joseph’s nodule. Int J Dermatol.

[21] Toyoda T, Suzuki H, Nakajima T (2018). Successful diagnosis of an occult fallopian tube carcinoma detected from the diaphragm metastasis. Gen Thorac Cardiovasc Surg.

[22] Kokcu A, Celik H, Kefeli M, Yucel I (2014). Co-existence of primary fallopian tube carcinoma and uterine carcinosarcoma. J Obstet Gynaecol.

[23] Mladenović-Segedi L (2009). Primary fallopian tube carcinoma. Med Pregl.

[24] Levine DA, Argenta PA, Yee CJ (2003). Fallopian tube and primary peritoneal carcinomas associated with BRCA mutations. J Clin Oncol.

[25] Aziz S, Kuperstein G, Rosen B (2001). A genetic epidemiological study of carcinoma of the fallopian tube. Gynecol Oncol.

[26] Tonin P, Moslehi R, Green R (1995). Linkage analysis of 26 Canadian breast and breast-ovarian cancer families. Hum Genet.

[27] Rose PG, Shrigley R, Wiesner GL (2000). Germline BRCA2 mutation in a patient with fallopian tube carcinoma: a case report. Gynecol Oncol.

[28] Pectasides D, Pectasides E, Economopoulos T (2006). Fallopian tube carcinoma: a review. Oncologist..

[29] Klein M, Rosen AC, Lahousen M, Graf AH, Rainer A (1999). Lymphadenectomy in primary carcinoma of the fallopian tube. Cancer Lett.

[30] Winter-Roach BA, Tjalma WA, Nordin AJ, Naik R, de Barros LA, Monaghan JM (2001). Inguinal lymph node metastasis: an unusual presentation of fallopian tube carcinoma. Gynecol Oncol.

[31] Deffieux X, Morice P, Thoury A, Camatte S, Duvillard P, Castaigne D (2005). Anatomy of pelvic and para-aortic nodal spread in patients with primary fallopian tube carcinoma. J Am Coll Surg.

[32] Gerolymatos A, Yannacou N, Bontis N, Chranioti S, Tsionis C, Mavrikios G (1993). Primary fallopian tube adenocarcinoma with metastasis to the uterine cervix. A case report. Eur J Gynaecol Oncol.

[33] Pradeep S, Kim SW, Wu SY (2014). Hematogenous metastasis of ovarian cancer: rethinking mode of spread. Cancer Cell.

[34] Stasenko M, Fillipova O, Tew WP (2019). Fallopian tube carcinoma. J Oncol Pract.

[35] Pectasides D, Pectasides E, Papaxoinis G (2009). Primary fallopian tube carcinoma: results of a retrospective analysis of 64 patients. Gynecol Oncol.

